# Feasibility of applying infrared thermal imaging for home monitoring of arthritis in children

**DOI:** 10.1186/s12969-025-01096-1

**Published:** 2025-07-31

**Authors:** Stephen Wong, Nivrutti Bhide, Erin Balay-Dustrude, Erin Sullivan, Joshua Scheck, Ian Muse, Kevin Cain, Debosmita Biswas, Savannah C. Partridge, Yongdong Zhao

**Affiliations:** 1 Pediatric Rheumatology, Department of Pediatrics, Seattle Children’s Hospital, University of Washington, 4800 Sand Point Way NE, Seattle, WA 98105 USA; 2 Center for Clinical and Translational Research, Seattle Children’s Research Institute, Seattle, WA USA; 3 Department of Statistics, School of Nursing, University of Washington, Seattle, WA USA; 4 Department of Radiology, University of Washington, Seattle, WA USA

**Keywords:** Juvenile idiopathic arthritis, Pediatric rheumatology, Knee, Infrared thermal imaging, Telemedicine

## Abstract

**Background:**

Telemedicine has improved access to pediatric rheumatology care. A disadvantage to using virtual modality for evaluation of children with arthritis is the lack of an in-person, hands-on physical exam. Thermal imaging has been studied in the clinical setting with promising results. This study aims to determine the feasibility of procuring at-home thermal imaging, measuring the variability of in-home skin temperature measurements over three consecutive days, and to compare these measurements at home to ones obtained in the clinic setting.

**Methods:**

Children with knee pain and/or swelling for a week or longer were enrolled and imaged with a smartphone-attached FLIR ONE PRO and Fluke handheld cameras followed by imaging with a FLIR camera at home for 3 consecutive days. Joint exam performed in the office was used as gold standard for joint assessment. A previously validated metric of temperature after within-limb calibration (TAWiC), defined as the temperature differences between the knee joint and ipsilateral mid-tibia, was used for all imaging studies.

**Results:**

Fifty-three patients were enrolled and thirty-eight completed the imaging acquisition at home with analyzable images. When evaluating images of the knee and mid-tibia regions, images collected at home compared to in-office demonstrated consistently lower absolute temperatures. However, the calibrated temperatures (TAWiC) of the anterior and lateral views of the knee showed mild to moderate correlation across 3 days between home-acquired images and office-acquired images (*r* = 0.58, 0.26, 0.24 and *r* = 0.36, 0.41, 0.42, respectively). The sensitivity and specificity of detecting arthritis of the knee using TAWiC adjustments from previously defined thresholds were similar regardless of the setting of image acquisition (0.44 and 0.79).

**Conclusions:**

This study demonstrates the feasibility of applying TAWiC for arthritis detection through a smartphone-based infrared thermal camera operated by families at home. Further investigation on a larger scale is needed prior to implementation of this process in the telemedicine setting.

**Supplementary Information:**

The online version contains supplementary material available at 10.1186/s12969-025-01096-1.

## Background

Juvenile idiopathic arthritis (JIA) is the most common rheumatologic disorder that presents to the pediatric rheumatology practice and may be more common than previously realized [1]. Knees are the most commonly affected joints [2]. Early diagnosis and aggressive treatment lead to improved outcome and higher rates of clinical remission [3]. With an overall shortage in practicing pediatric rheumatologists in the USA compounded by the heterogeneous distribution favoring urban settings for pediatric rheumatology practices, families have to travel long distances to receive care for their children who have arthritis [4]. Access to care is one of the most important barriers to early diagnosis and treatment. With advances in telemedicine spurred by the COVID-19 pandemic, pediatric rheumatology telemedicine visits have led to improved access for families of children with JIA [5]. However, diagnosis of JIA can be hindered by the lack of an in-person, hands-on physical exam by a trained pediatric rheumatologist. Some pediatric rheumatologists have been performing virtual pediatric musculoskeletal exams, the Video pediatric Gait, Arms, Legs, and Spine (V-pGALS) assessment, which has been shown to be reliable, acceptable, and practical [6]. When virtual physical exams are equivocal, further imaging such as contrast-enhanced magnetic resonance imaging (MRI) and musculoskeletal ultrasounds are often required. These tests can be costly and cannot be performed at the visit concurrently. Non-contact infrared thermal imaging of the joints can be used to evaluate for high thermographic temperatures and has been shown to correlate with positive power doppler signal on bedside musculoskeletal ultrasound in adults with knee arthritis [7]. We have previously demonstrated that the adjusted temperature, Temperature After Within-limb Calibration (TAWiC), increased the accuracy of arthritis detection by > 30% [8]. Furthermore, our group has shown that TAWiC derived from images acquired with the handheld high-resolution thermal camera (FLUKE Ti32) is similar to that from the smartphone-based low-resolution thermal camera in pediatric patients (FLIR ONE PRO), which created the opportunity for applying this technology as an adjunct tool for patients to monitor their joint health at home [9]. A smartphone-attached camera is affordable, small, and can be shipped to families before their telemedicine visit. With appropriate and standardized training, families can utilize this non-invasive device to aid in the monitoring of inflammatory arthritis. Implementation and accurate interpretation of thermal imaging in the home setting may assist physicians with decision making during telemedicine encounters. The objective of this study is to determine the feasibility of collecting analyzable family-procured infrared thermal images using FLIR ONE PRO in home setting, measuring the variability of in-home skin temperature measurements over three consecutive days, and to compare these measurements at home to ones obtained in the clinic setting.

## Methods

Institutional review board (IRB) approval (#1383) was obtained from Seattle Children’s Hospital. Patients with knee pain and/or swelling for at least a week between the ages of 2 and 18 years, were consented and enrolled. Children with fever, potential confounding cutaneous infections or inability to pose for static imaging were excluded.

### Image acquisition

As previously described [8], infrared thermal imaging of the lower limbs from four views (anterior, posterior, medial and lateral) was performed using FLIR™ ONE PRO cameras (Teledyne FLIR LLC, Wilsonvillem, IR) with 19,200 pixels and a detection range from − 20 to 400 °C, sensitivity ≤ 0.07 °C (70 mK) and a Fluke™ TiR32 Thermal Imager (Fluke Inc., Everett, WA) with 76,800 pixels (320 × 240) (detection range − 20 to 150 °C, sensitivity ≤ 0.04 °C) by research team at the office after enrollment. Families were then trained in how to take images using their own phones and provided FLIR cameras in the office. An image upload link was provided for them to send collected images from home to our team via REDCap. A video instruction was also shared with family as a reference.

### Analysis of thermal images

**Fig. 1:**
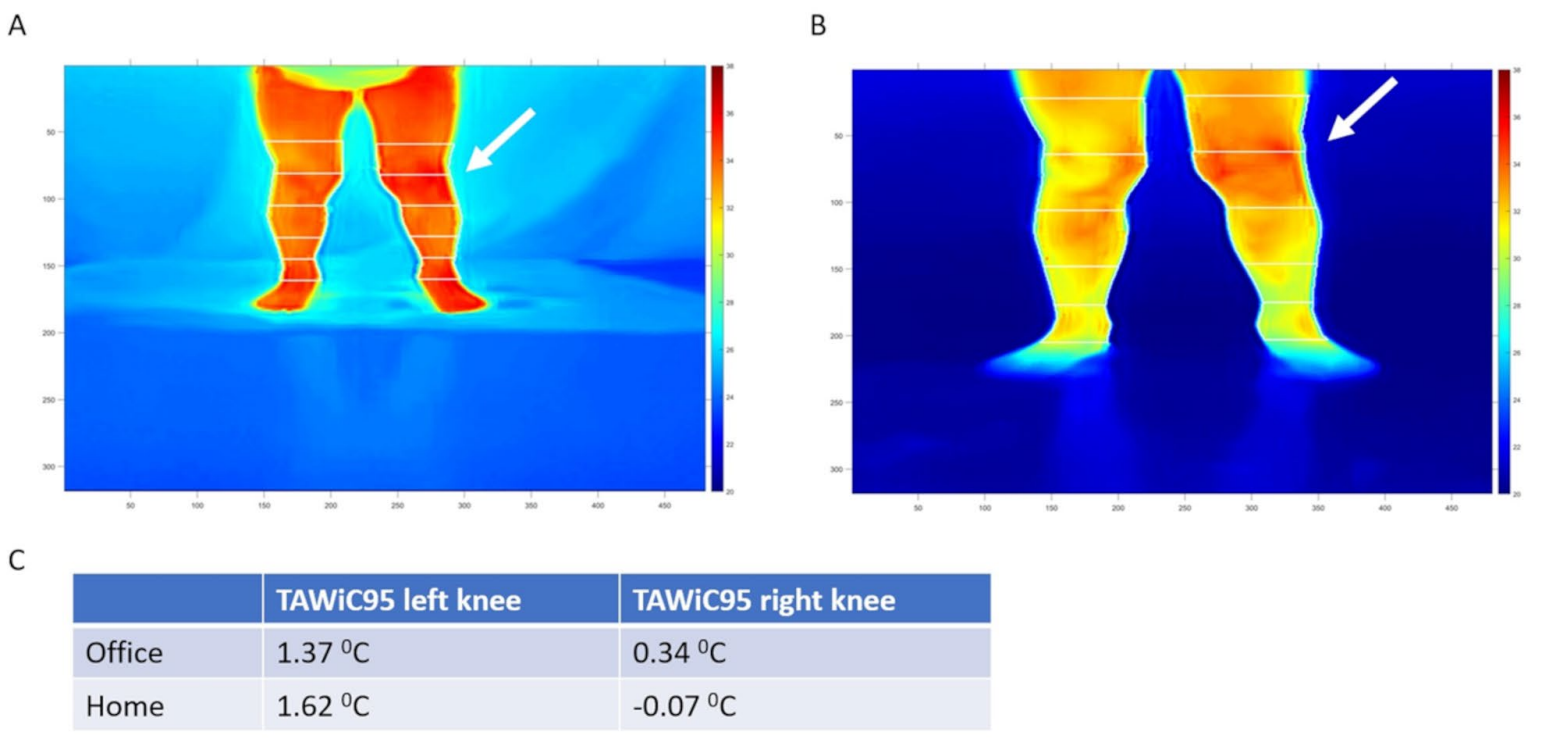
Representative thermal images obtained from office (**A**) and home (**B**). TAWiC95 of both knees were reported in panel **C**. Thermal imaging showed elevated temperatures within left knee (white arrows) which was confirmed as arthritis by in-person and virtual physical exam

The spatial and temperature data from infrared thermal images were exported from FLIR software into csv files. Data were then analyzed using customized semi-automated software developed in MATLAB^®^ (Mathworks, Natick, MA) as previously reported [8]. The thermal data were imported into MATLAB^®^ followed by a threshold setting to filter out the background environment pixels. Crosshairs were then placed at the level of knee and ankle to define the length of lower leg which was further divided into three segments equally along the long axis. Another segment of the same length was added above the knee level and merged with the segment immediately below to define the ROI for the knee. Mean and 95th greatest temperatures were reported for each segment and the TAWiC of the knee was calculated by subtracting the corresponding mean or 95th temperatures in the mid segment of lower leg from that of the knee ROI (Fig. 1).

### Demographic, clinical and laboratory data collection

Demographic information including gender, age, and clinical data including body height, weight, oral or temporal temperatures were collected in all subjects. The presence or absence of joint swelling, pain, or warmth were recorded. Laboratory data were also collected if available.

### Data analysis

Histograms were examined for outliers and non-normality. Demographic variables were summarized for the population. Absolute and TAWiC temperatures are summarized by location and day (office, at home days 1, 2, 3). Three images per view were obtained per participant at each time point and the mean of these three values was computed to represent the time point. Within-patient standard deviation (representing the standard deviation of three repeated images in office, and at home on each of days 1–3) was compared between the office and at home settings using generalized estimating equations to account for correlation among multiple observations per patient. Pearson’s correlation coefficients were calculated to compare at home measurements to in office measures. Mean differences with 95% confidence intervals were calculated by day, comparing each day’s at home measurement to the in-office measurement to assess daily patterns using generalized estimating equations methods, accounting for within patient correlation between two legs measured (left and right). Analyses were done separately for each view (anterior, lateral, medial, posterior). Sensitivity and specificity of detecting knee arthritis by day and location were calculated with 95% confidence intervals using derived thresholds. A p-value below 0.05 was considered statistically significant. All analyses were done using SAS 9.4 (Cary, NC).

## Results

### Demographic characteristics

Fifty-three patients were enrolled in the study and thirty-eight completed the imaging acquisition at home with analyzable images. Thirteen families did not upload the requested images. Two participants were excluded due to the diagnosis of systemic lupus erythematosus or received intraarticular steroid injection during the initial office visit. Baseline characteristics of participants who completed the home acquisition protocol are summarized in Table 1. Most participants were females (72%), and the mean age was 11.8 years old. The majority of participants did not have a JIA diagnosis, or a diagnosis was unknown at time of image collection (68%, *n* = 26). Those with a JIA diagnosis were largely oligoarticular (18%, *n* = 7), with other JIA categories less represented (3%, *n* = 1).

**Table 1:** Baseline characteristics of children with at home thermal imaging data

Characteristics	All Patients *N* = 38
Age at enrollment (years) *	11.8 (4.0)
Female	27 (71.1%)
Weight (kg)*	50.0 (22.6)
Height (cm)*	149.0 (22.3)
BMI*	21.4 (5.4)
Oral temperature (^0^C) *N* = 32	37.0 (0.3)
**ILAR category**	
Persistent oligoarticular	7 (18.4%)
Extended Oligo	1 (2.6%)
RF + poly	1 (2.6%)
RF- poly	1 (2.6%)
ERA	1 (2.6%)
Psoriatic	1 (2.6%)
Not JIA/Unknown	26 (68.4%)
**Laboratory findings**	
ANA positive	12/30 (40.0%)
RF positive	1/26 (3.8%)
CCP positive	3/25 (12.0%)
HLA-B27 positive	2/23 (8.7%)
Erythrocyte sedimentation rate, mm/h (normal 0–20) *N* = 30**	3 (2–7)
**Treatment at study entry**	
NSAIDs	29 (76.3%)
DMARD	3 (7.9%)
Biologic	1 (2.6%)
Systemic glucocorticoids	0 (0.0%)
*Mean (SD)	
**Median (IQR)	

### Feasibility of home thermal image acquisition

The majority of the study participants (75%, 40 out of 53) were able to capture at home thermal images suitable for analysis and detection of arthritis with thirty-five participants collecting all three days, and the remaining three collecting at least one day. This indicates the feasibility of collection of thermal images with appropriate training. One common difficulty encountered in home acquisition was the inclusion of extraneous objects in the acquired images such as pets, which may have affected temperature measurement accuracy.

### Comparability of the images acquired at home and in-office

Thermal measurements obtained by FLIR ONE PRO were not significantly different from those obtained from FLUKE Ti32 imager in the office. With the in-home acquisition using only a FLIR camera, image analysis demonstrated that 95th absolute and mean absolute temperature measurements decreased on subsequent home imaging days compared to the measurements taken in the office. Mean TAWiC measurements showed a similar pattern, while 95th TAWiC measurements initially showed a decrease, followed by an increase by day 3 (Tables 2 and 3).

**Table 2:** The comparison of the 95th absolute temperature and temperature after Within-limb calibration (TAWiC) of the knee joints derived from images obtained in office and at home

	Office FLUKE	Office FLIR	Day 1 at home with FLIR		Day 2 at home with FLIR		Day 3 at home with FLIR	
Total number of joints (*N* = 76)	Mean (SD) Temperature	Mean (SD) Temperature	Mean (SD) Temperature	Correlation With In Office	Mean (SD) Temperature	Correlation With In Office	Mean (SD) Temperature	Correlation With In Office
**Knee**								
95th absolute (Anterior)	33.71 (1.23)	31.63 (2.03)	29.51 (2.42)	0.08	28.97 (3.1)	-0.02	27.94 (2.93)	0.21
95th absolute (Lateral)	33.34 (1.13)	33.00 (1.38)	30.44 (2.22)	0.20	30.2 (2.19)	0.20	30.04 (2.11)	0.19
95th absolute (Medial)	33.43 (1.15)	33.11 (1.37)	30.58 (2.31)	0.31*	30.28 (2.28)	0.22	30.13 (2.24)	0.17
95th absolute (Posterior)	33.74 (1.04)	33.93 (1.28)	30.96 (2.17)	0.33*	30.9 (2.6)	-0.02	30.41 (2.43)	0.01
95th TAWiC (Anterior)	0.04 (0.71)	0.09 (0.71)	0.03 (0.69)	0.58*	0.19 (0.73)	0.26*	0.25 (0.81)	0.24*
95th TAWiC (Lateral)	0.26 (0.57)	0.30 (0.59)	0.43 (0.90)	0.36*	0.48 (0.72)	0.41*	0.52 (0.72)	0.42*
95th TAWiC (Medial)	0.50 (0.71)	0.49 (0.75)	0.54 (0.71)	0.03	0.47 (0.64)	0.09	0.69 (0.68)	0
95th TAWiC (Posterior)	1.50 (0.42)	1.36 (0.38)	1.39 (0.58)	0.10	1.2 (0.54)	0.12	1.46 (0.56)	0.03
**Mid tibia**								
95th absolute (Anterior)	33.68 (1.27)	31.54 (2.23)	29.48 (2.12)	-0.06	28.78 (2.92)	-0.11	27.68 (2.94)	0.28*

* *p* < 0.05

**Table 3:** The comparison of the mean absolute temperature and temperature after Within-limb calibration (TAWiC) of the knee joints derived from images obtained in office and at home

	Office FLUKE	Office FLIR	Day 1 at home with FLIR		Day 2 at home with FLIR		Day 3 at home with FLIR	
Total number of joints (*N* = 76)	Mean (SD) Temperature	Mean (SD) Temperature	Mean (SD) Temperature	Correlation With In Office	Mean (SD) Temperature	Correlation With In Office	Mean (SD) Temperature	Correlation With In Office
**Knee**								
Mean absolute (Anterior)	32.27 (1.30)	30.28 (2.17)	28.19 (2.58)	0.08	27.61 (3.04)	-0.05	26.56 (2.88)	0.2
Mean absolute (Lateral)	31.99 (1.19)	31.71 (1.37)	29.13 (2.27)	0.2	28.94 (2.2)	0.13	28.75 (2.03)	0.17
Mean absolute (Medial)	32.14 (1.21)	31.9 (1.41)	29.26 (2.35)	0.26*	29.09 (2.29)	0.15	28.87 (2.15)	0.19
Mean absolute (Posterior)	32.22 (1.14)	32.49 (1.38)	29.5 (2.26)	0.31*	29.46 (2.69)	-0.08	28.96 (2.46)	0.03
Mean TAWiC (Anterior)	-0.26 (0.57)	-0.29 (0.68)	-0.33 (0.69)	0.49*	-0.14 (0.71)	0.31*	-0.13 (0.72)	0.33*
Mean TAWiC (Lateral)	-0.06 (0.46)	-0.06 (0.47)	0.05 (0.62)	0.56*	0.13 (0.58)	0.49*	0.20 (0.55)	0.32*
Mean TAWiC (Medial)	0.18 (0.53)	0.25 (0.57)	0.18 (0.49)	0.19	0.25 (0.61)	0.1	0.38 (0.6)	0.14
Mean TAWiC (Posterior)	0.95 (0.41)	0.96 (0.36)	0.97 (0.56)	0.22	0.85 (0.49)	0.19	1.11 (0.51)	0.16
**Mid tibia**								
Mean absolute (Anterior)	32.53 (1.28)	30.58 (2.17)	28.51 (2.19)	-0.05	27.75 (2.89)	-0.13	26.69 (2.91)	0.3*

* *p* < 0.05

Correlation between the measurements derived from the images acquired in the office and those at home was overall very weak to moderate using Pearson’s correlation coefficients, ranging from − 0.06 to 0.58, with the highest correlation seen with mean and 95th anterior TAWiC. For example, 95th anterior TAWiC measurements demonstrated 0.58, 0.26 and 0.24 correlation with office measurements on day 1, 2, and 3, respectively. Correlation generally down trended over the subsequent days of home measurements. Average within patient standard deviation, representing the precision of multiple images taken consecutively in each setting, was consistently higher in the at home setting (ranging from 0.11 to 0.43) than in the office (ranging from 0.07 to 0.16). This difference was statistically significant at an alpha = 0.05 for all absolute temperatures, however the difference was smaller for TAWiC measurements and was not significant for either mean or 95th percentile TAWiC measured via the anterior or posterior views, adding to the evidence of TAWiC’s robust utility (supplemental table).

**Fig. 2:**
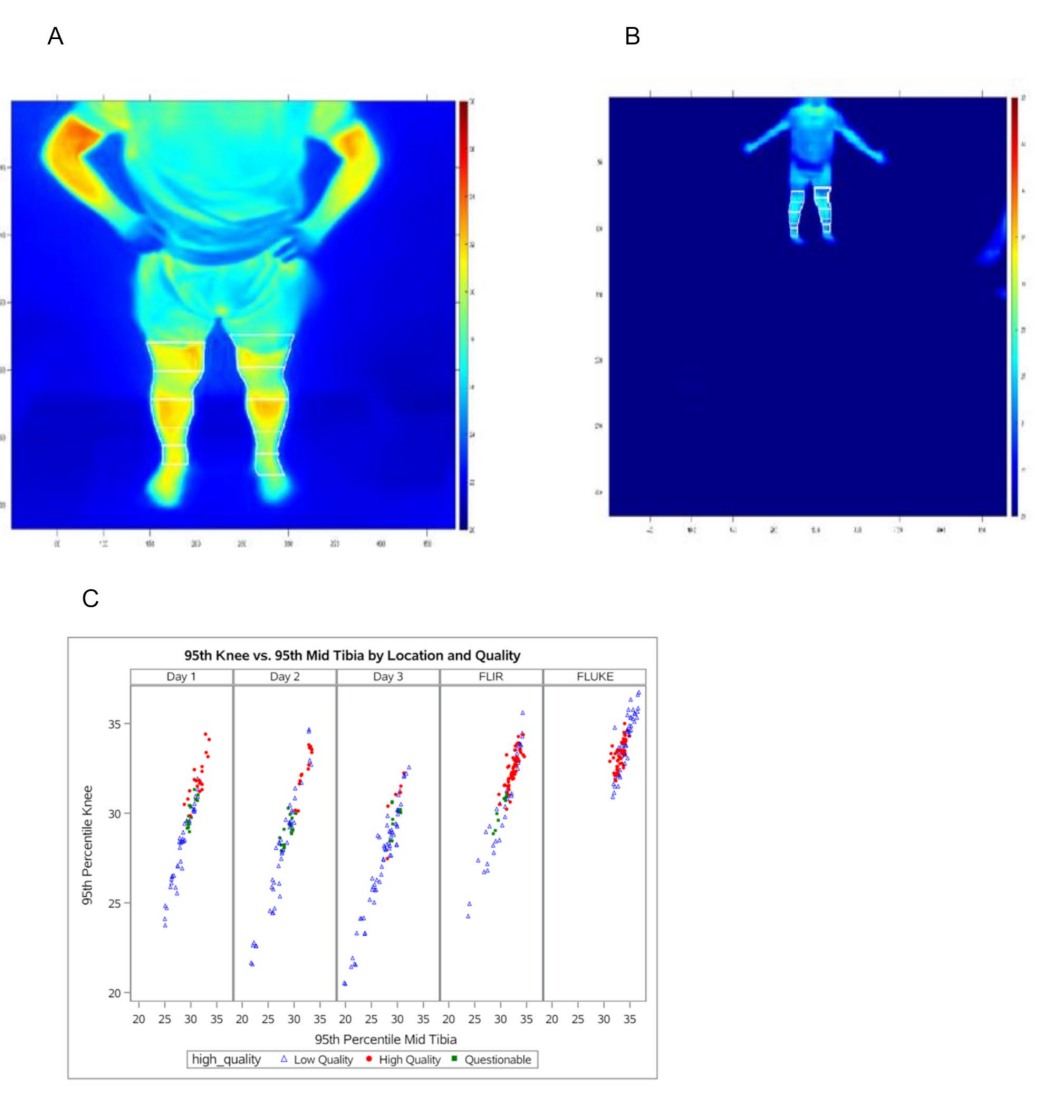
Representative quality of home images (**A** and **B**) along with the scattered plot of thermal imaging quality by location (**C**). Review of home thermal images were noted as high quality (**A**) and low quality (**B**)

It should also be noted that images were assessed as either high quality, low quality or questionable quality by research staff depending on body and ambient temperature readings **(**Fig. 2**)**. Office-obtained imaging either using FLUKE Ti32 camera or FLIR ONE PRO camera were more likely to obtain images that were of high quality at 54% and 58%, respectively. On the other hand, thermal images obtained at home were only rated as high quality in 29%, 18%, and 8% on days 1, 2, and 3, respectively **(**Fig. 2**)**.

### Sensitivity and specificity for arthritis detection

Sensitivity and specificity of detecting arthritis were calculated by using the 95th TAWiC anterior view for office and home acquired images **(**Table 4**)**. Sensitivity was comparable among home and office measurements, ranging from 44% in the office to up to 56% at home on day three. Specificity was notably higher than sensitivity, ranging from 79% in the office, up to 85% on home imaging on day one.

**Table 4:** In-Office and At-Home sensitivity and specificity for detection of arthritis using the 95th TAWiC anterior view measurement

Knee	Office FLUKE	Office FLIR	Day 1 Home	Day 2 Home	Day 3 Home
Total number of patients with analyzable image	37	38	35	36	37
Number of patients with history of or newly diagnosed JIA	12	12	12	12	11
Number of patients without history of JIA	25	26	23	24	26
Number of patients with active knee arthritis	7	7	7	7	6
Number of knees with active arthritis	9	9	9	9	8
Sensitivity (95% CI)	44.4% (12.0 − 76.9%)	44.4% (12.0 − 76.9%)	44.4% (12.0 − 76.9%)	44.4% (12.0 − 76.9%)	62.5% (29.0 − 96.1%)
Specificity (95% CI)	84.6% (75.8 − 93.4%)	79.1% (67.4 − 88.1%)	83.6% (74.3 − 92.9%)	71.4% (58.7 − 82.6%)	71.2% (60.3 − 82.1%)

TAWiC: Temperature After Within-limb Calibration

Footnote: detection of arthritis was determined using the previously defined threshold of 0.54 from 95th TAWiC anterior view

## Discussion

To the best of our knowledge, this is the first study that has attempted to utilize infrared thermal imaging in the home setting. There have been prior studies that have found good correlation of thermal imaging using a FLUKE Ti32 camera and a smartphone-attached FLIR ONE PRO camera [7], [8], [9]. These studies were performed in an office setting by trained research staff. We understand this technology may aid with detecting arthritis in all clinic environments, but hypothesized its use in virtual, telemedicine clinic visits may be just as, if not, more important given the lack of a hands-on exam by a trained rheumatologist. The results of this study demonstrated that most families (40/53) were able to complete an online tutorial, successfully procure at least one analyzable images and upload these images online for remote interpretation. Though all families were assisted to make sure the FLIR ONE software was installed correctly on their smartphones, the upload of images to our database required scanning a QR code and following online prompts. We made up to 3 attempts to contact each family and those who did not upload images did not provide reasons for the inability to upload images. It is also unclear why some families were able to upload images from 1 to 2 days but not all 3 days at home. Feedback from families regarding the realistic barriers is essential for a better user interface design to improve the engagement and usage. It would seem though, that most home thermal images, though analyzable, were of questionable or low quality, which could be due to user error but was more likely due to an unstandardized approach for home procurement. This unstandardized approach may have led to some imaging results that were adversely affected by having pets and highly thermally-conductive metal objects (e.g., Door knobs, lamps, etc.) positioned near the joint region of interest. A lack of standardized home ambient temperatures may also have negatively affected the quality of home thermal imaging. Calibration of the FLIR ONE PRO at each home prior to image acquisition may also improve the quality of the thermal images and its use to detect arthritis. Development of future standardized protocols in a future project should identify these background noises and factors to optimize accurate thermal imaging acquisition, thus, improving the rate of procured, high quality, analyzable images.

Considering the reliability of acquiring images at home, the family-obtained images from a FLIR ONE PRO infrared thermal camera in a home setting displayed moderate correlation with images obtained by research staff in the office setting when comparing TAWiC values from anterior and lateral images. Furthermore, sensitivity and specificity tests for detection of arthritis were similar between the home and office-acquired images. On the other hand, correlation of absolute temperature was weak. This could be due to trends showing the absolute temperatures of knees and mid-tibia (mean and 95th percentile) were consistently lower at home compared to those that were acquired in the office, although TAWiC values were similar. These results suggest that TAWiC is more robust than the absolute temperature in detecting arthritis, which is independent of the mild fluctuation of the environmental ambient temperatures between different homes and office locations. Caution should be used in interpreting the results of this study and comparing it to validated in-office data since many of the in-home acquired thermal images were of questionable or low quality. Also, further caution should be made when interpreting the reported sensitivity and specificity values for in-home thermal images since all in-home images were obtained days after the clinical examination that determined if the study subject had active knee arthritis. In order to calculate the in-home thermal imaging sensitivity and specificity, the assumption was made that there was no change in arthritis status between the day of the clinic visit and days of in-home thermal image acquisition.

The possible incongruity of the absolute temperatures could be due to variations of in-home temperatures with an average home temperature being lower than the office temperature, although home temperature settings or measurements were not obtained as part of this study to confirm this hypothesis. However, the TAWiC measurement overcame the variation of absolute temperature and led to robust determination of arthritis comparably at home as in the office, even across user variability. Other factors that may lead to fluctuations in absolute or TAWiC temperatures could include circadian rhythm, and circamensal rhythms [10]. Body temperatures tend to be the highest during mid-afternoon and fall in the evening. If families are obtaining temperature readings after school, it may explain the lower mean absolute temperatures obtained in the home setting. Another factor that could explain the higher skin temperatures in the office setting could be due to stress, as it has been shown that core body temperatures and skin temperatures rise in certain body regions when study participants are exposed to stress [11]. Regional changes in the temperature over joints secondary to psychological stress have not been specifically studied, and no formal stress testing was done during the study clinic visit to explore this possibility. Lastly, the duration of resting prior to imaging, and administration of medications with antipyretic properties could also affect absolute temperature measurements. Instructions for families to collect images at a consistent time of day after adequate resting, preferably in the morning may mitigate this issue in future studies when developing future standardized protocols.

By demonstrating families’ ability to acquire thermal images of the joints at home, this study added strong evidence as a proof of concept towards future implementation. Further research should focus on removing the variables that may lead to inconsistencies between home and office thermal images. The significant drop in quality of thermal images from home setting demonstrated the need for in-depth training and a standardized protocol, potentially through real time feedback via web portal or smartphone application. Examples include having families acquire thermal images immediately after research staff have imaged the joints in the office environment to confirm the accuracy of the family-obtained temperatures (evaluating inter-user reliability), providing a mini-kit including a neutral backdrop, or developing protocols that standardize or account for home temperature and acquisition time differences.

## Conclusions

This study demonstrates the feasibility of using thermal imaging in the home setting, with most families successfully acquiring and uploading analyzable thermal images. There was moderate correlation between home vs. office TAWiC measurements of the anterior leg, though the study protocol was not rigorously developed to limit confounding variables and barriers. Future studies using standardized protocols for home thermal image acquisition will be necessary to improve accuracy and precision of thermal images obtained by a smartphone-based FLIR ONE PRO in the home setting. Moreover, a larger scale study is needed to further validate our findings prior to implementation in telemedicine practice.

## Electronic supplementary material

Below is the link to the electronic supplementary material.

Supplementary Material 1

## Data Availability

No datasets were generated or analysed during the current study.

## Abbreviations

JIA: Juvenile idiopathic arthritis
ROIs: Regions of interest
TAWiC: Temperature after within-limb calibration
V-pGALS: Video pediatric Gait, Arms, Legs, and Spine
MRI: Magnetic resonance imaging
SCRI: Seattle Children’s Research Institute
IRB: Institutional review board
