# Supplementary material for: Feasibility of applying infrared thermal imaging for home monitoring of arthritis in children
Source: Pediatr Rheumatol Online J. 2025 Jul 31;23:84. doi: 10.1186/s12969-025-01096-1 (PMC12312333; doi:10.1186/s12969-025-01096-1)
Supplement: Supplementary file 1 — Supplementary Material 1 [file 12969_2025_1096_MOESM1_ESM.docx]

Supplemental Materials

A training link to an approximately 8-minute long instructional video to be provided to parents/participants:

<https://youtu.be/qGZcHXPgy3E>

**Supplemental Table.** Comparison of Within Patient Standard Deviation of Replicate Images by Location

|  | **In Office FLIR Within Patient Standard Deviation** | **Day 1 Within Patient Standard Deviation** | **Day 2 Within Patient Standard Deviation** | **Day 3 Within Patient Standard Deviation** | **p-value *** |
| --- | --- | --- | --- | --- | --- |
| **Knee** |  |  |  |  |  |
| **95th absolute (Anterior)** | 0.16 (0.45) | 0.40 (0.86) | 0.38 (0.55) | 0.43 (0.92) | 0.0477 |
| **95th absolute (Lateral)** | 0.09 (0.08) | 0.19 (0.40) | 0.24 (0.39) | 0.20 (0.35) | < .0001 |
| **95th absolute (Medial)** | 0.08 (0.08) | 0.17 (0.45) | 0.23 (0.38) | 0.21 (0.32) | 0.0003 |
| **95th absolute (Posterior)** | 0.08 (0.08) | 0.18 (0.30) | 0.16 (0.23) | 0.11 (0.15) | 0.0049 |
| **95th TAWiC (Anterior)** | 0.08 (0.06) | 0.07 (0.06) | 0.11 (0.10) | 0.12 (0.20) | 0.1399 |
| **95th TAWiC (Lateral)** | 0.06 (0.05) | 0.09 (0.10) | 0.08 (0.06) | 0.07 (0.08) | 0.0271 |
| **95th TAWiC (Medial)** | 0.07 (0.04) | 0.09 (0.09) | 0.10 (0.12) | 0.08 (0.07) | 0.0501 |
| **95th TAWiC (Posterior)** | 0.08 (0.06) | 0.08 (0.06) | 0.09 (0.08) | 0.08 (0.07) | 0.5356 |
| **Mean absolute (Anterior)** | 0.15 (0.43) | 0.38 (0.84) | 0.39 (0.55) | 0.42 (0.90) | 0.0354 |
| **Mean absolute (Lateral)** | 0.07 (0.07) | 0.17 (0.44) | 0.21 (0.38) | 0.19 (0.34) | 0.0002 |
| **Mean absolute (Medial)** | 0.07 (0.07) | 0.16 (0.44) | 0.21 (0.38) | 0.19 (0.33) | 0.0004 |
| **Mean absolute (Posterior)** | 0.07 (0.07) | 0.16 (0.28) | 0.16 (0.25) | 0.10 (0.15) | 0.0037 |
| **Mean TAWiC (Anterior)** | 0.05 (0.04) | 0.06 (0.08) | 0.08 (0.07) | 0.07 (0.12) | 0.1094 |
| **Mean TAWiC (Lateral)** | 0.04 (0.03) | 0.06 (0.06) | 0.06 (0.06) | 0.06 (0.05) | 0.0081 |
| **Mean TAWiC (Medial)** | 0.04 (0.03) | 0.06 (0.08) | 0.06 (0.07) | 0.06 (0.05) | 0.0097 |
| **Mean TAWiC (Posterior)** | 0.05 (0.04) | 0.06 (0.05) | 0.07 (0.06) | 0.06 (0.06) | 0.1108 |
| **Mid tibia** |  |  |  |  |  |
| **95th absolute (Anterior)** | 0.16 (0.45) | 0.38 (0.84) | 0.37 (0.53) | 0.38 (0.79) | 0.0505 |
| **TAWiC: Temperature After Within-limb Calibration, ROI: region of interest,** | | | | |  |
| **Data availability (patients with at least one image): 35/38 on Day 1, 36/38 on Day 2, 37/38 on Day 3** | | | | | |
| *** Comparing standard deviation observed from in-home images to that observed in office using generalized estimating equations methods accounting for repeated measures within patients across multiple days on two legs** | | | | | |
